# Sleep and False Memory Production: The Modulating Role of Immediate Testing and Type of Retrieval

**DOI:** 10.1111/jsr.70051

**Published:** 2025-03-21

**Authors:** Francesca Conte, Serena Malloggi, Oreste de Rosa, Gianluca Ficca, Fiorenza Giganti, Nicola Cellini

**Affiliations:** ^1^ Department of Psychology University of Campania L. Vanvitelli Caserta Italy; ^2^ Department NEUROFARBA University of Florence Florence Italy; ^3^ Department of General Psychology University of Padova Padova Italy; ^4^ Human Inspired Technology Center University of Padova Padova Italy

**Keywords:** Deese‐Roediger‐McDermott paradigm, false memory formation, sleep‐related memory consolidation

## Abstract

Data on the effect of sleep on false memories are mixed. Here we address two methodological issues which could explain these inconsistencies: the use of free recall vs. recognition tasks and the absence of immediate testing. In a mixed design, 8 word lists from the Deese‐Roediger‐McDermott task were administered to a Recall (*n* = 20) and a Recognition Group (*n* = 20) at 9 AM (‘Wake’ condition) or 9 PM (‘Sleep’ condition). Immediate Testing (IT) followed the presentation of 4 out of 8 lists. Delayed Testing was performed 12 h later. A week later, participants underwent the other condition with 8 different lists. In both groups: IT performance was similar between conditions; veridical recalls were facilitated by Sleep and by IT irrespectively of condition. False memories were enhanced in Sleep vs. Wake for non‐tested lists only in the Recall Group. IT affected delayed false recognition regardless of the condition, whereas its effects on delayed recall differed between conditions: in Sleep, the false recall rate was similar between tested and non‐tested lists, whereas in Wake, it was higher for tested than non‐tested lists. Our results confirm that sleep enhances false recalls but not false recognitions when memory is tested directly after the retention interval. Moreover, immediate testing enhances veridical memory independently of condition, whereas its effect on false recall depends on the behavioural state in which retention occurs. Therefore, immediate testing could be usefully introduced in standard sleep–memory studies, while its use should be carefully evaluated in studies on sleep and false memories.

## 1 Introduction

Several studies over the past 20 years have addressed the role of sleep‐related memory consolidation in false memory formation (for a review, see Newbury and Monaghan 2019), mostly by comparing false memory production after a retention interval spent asleep or awake. These studies have yielded mixed results, with some reporting enhanced false memory production after sleep relative to wake (Payne et al. 2009; Diekelmann et al. 2010; McKeon et al. 2012; Malloggi et al. 2023) and others observing a reduction in false memories after sleep (Fenn et al. 2009; Lo et al. 2014) or even no differences between conditions (Diekelmann et al. 2008).

These controversial findings can be explained by the methodological inconsistencies between studies (Newbury and Monaghan 2019), mainly linked to differences in the tasks used to evaluate memory performance. Indeed, most studies addressing sleep and false memories have employed the Deese–Roediger–McDermott paradigm (DRM; Deese 1959; Roediger and McDermott 1995), the task most frequently used by psychologists of memory to investigate false memory formation in laboratory settings (Pardilla‐Delgado and Payne 2017; Gallo 2010). However, the adaptation of the DRM to sleep research designs has entailed a significant variability, among sleep studies, in task details and procedures.

In its original version, the DRM paradigm consists of the administration of an immediate free recall test followed by a recognition test on lists of words that are semantically associated with an unstudied critical word (e.g., *door*, *glass*, *pane*, *shade*, *ledge*, *sill*, *house*, *open*, *curtain*, all related to *window*). This task reliably produces high rates of confident false memories and false recognitions for unstudied critical words (Roediger and McDermott 1995). In sleep studies, the memory for the lists is tested, either through a free recall or a recognition task, after a retention period spent in sleep or wake. As highlighted by Newbury and Monaghan (2019), several DRM characteristics could modulate the influence of sleep on the production of false memories. Among the possible factors (e.g., the number of lists and words composing each list, the presentation procedure, the degree of emotionality of the lists, etc.), the nature of the task adopted for testing (i.e., free recall or recognition) appears to be the main moderator of the effect of sleep on false memories. Specifically, the studies employing free recall procedures mostly reported an increase in false recalls after sleep compared to wake (e.g., Payne et al. 2009; Diekelmann et al. 2010; McKeon et al. 2012; Mak et al. 2023; Malloggi et al. 2023), whereas research conducted with recognition tasks did not reveal differences between sleep and wake conditions (e.g., Diekelmann et al. 2008; Pardilla‐Delgado and Payne 2017; Malloggi et al. 2023) or even showed reductions in false recognitions after sleep compared to wake intervals (Fenn et al. 2009; Lo et al. 2014).

To date, as far as we are aware, only Pardilla‐Delgado and Payne (2017) have addressed the effect of sleep on false memories by employing a free recall and a recognition task in the same study. In line with previous literature, they observed that the retention period spent asleep promoted false recalls but did not exert an effect on recognition performance. Still, as noted by the same authors, the latter finding is confounded by the fact that, in their study, the free recall task always preceded the recognition task.

Another critical methodological aspect concerning the studies in this field is the evaluation of baseline measures of memory performance. Indeed, none of these studies (except Payne et al. 2009; see below) have assessed baseline DRM performance through an immediate recall or recognition test; rather, memory performance has been directly tested after the retention interval spent in sleep or wake. However, as in classical sleep‐memory studies, the evaluation of baseline performance is critical in order to exclude that performance observed at delayed testing depends on the effectiveness of encoding rather than specifically on consolidation processes that occurred over the retention interval (see Conte and Ficca 2013; Németh et al. 2024). In other words, without a baseline measure of false memory production, it is impossible to determine whether false memories produced after sleep or wake reflect the consolidation of these memories produced at encoding or the emergence of ‘new’ false memories over the retention interval. In addition, false memory performance at immediate testing may represent a useful index of individual propensity to produce false memories.

Payne et al. (2009) tried to address this issue through a between‐subjects designed study. In a DRM paradigm, an immediate free recall test was administered to a control group, while the experimental groups (Sleep and Wake groups) performed, as in the previously mentioned studies, the free recall test directly after a retention interval spent asleep or awake. Veridical recall deteriorated in both experimental groups relative to control participants' performance (though deterioration was significantly lower in the Sleep group); false recalls, instead, appeared reduced in the Wake group and preserved in the Sleep group. To the best of our knowledge, no other published study has addressed this issue.

Furthermore, the effects of immediate testing on delayed performance after sleep or wake may be worth investigating per se. Indeed, since immediate testing enhances the accessibility of memory traces at a subsequent task (Karpicke and Smith 2012), it could be the case that sleep differentially consolidates memory traces that have been encoded at different depths (see Conte and Ficca 2013, for a review), with different effects also on false memories production.

Here we address these two methodological issues, i.e., the type of test (recall vs. recognition) and the role of immediate testing, through a mixed‐design study aimed at evaluating false memory production at the DRM paradigm after a retention interval spent in sleep vs. wake. Participants are randomly assigned to two groups, differing in the nature of the task administered at immediate and delayed testing, i.e., a free recall and a recognition task. Regardless of the type of task, all participants are administered an immediate test on only half of the presented lists, in order to assess: (a) the proportion of false memories produced at delayed testing relative to immediate (baseline) performance in both conditions, (b) the possibility that the immediate testing procedure affects, per se, delayed false memory production, with possible differences between the sleep and wake conditions.

This procedure allows us to address several research questions: (a) does sleep promote delayed false memory formation only when retrieval is tested through free recall rather than recognition (as suggested by previous literature)? (b) is false memory production after sleep compared to wake dependent on offline memory processes going on during the retention interval rather than on differences at encoding? (c) how would the introduction of immediate testing, aimed to assess baseline performance at the DRM, affect delayed performance after a retention interval spent in sleep or wake?

## 2 Materials and Methods

### 2.1 Participants

Participants were recruited among university students who were screened through a set of ad hoc questions administered online through the Google Form platform, aimed at collecting demographic data and assessing general medical conditions and health habits, as well as the presence of psychiatric disorders and sleep problems. The questionnaire also included a set of standardised instruments: the Pittsburgh Sleep Quality Index (PSQI; Italian version from Curcio et al. 2013) to assess sleep quality, the Insomnia Severity Index (ISI; Italian version from Castronovo et al. 2016) to assess the presence of insomnia symptoms, the Beck Depression Inventory‐II (BDI‐II; Italian version from Sica and Ghisi 2007) and the Beck Anxiety Inventory (BAI; Italian version from Sica and Ghisi 2007) to assess depressive and anxiety symptoms, respectively. We also assessed circadian preferences using the Morningness‐Eveningness Questionnaire Reduced version (MEQr; Italian version from Natale et al. 2006).

Inclusion criteria were: age 18–35; absence of any relevant somatic or psychiatric disorder; no history of drug or alcohol abuse; absence of sleep disorders and any sleep apnoea or respiratory symptoms; having a regular sleep–wake pattern (e.g., individuals with irregular study or working habits such as shift‐working were excluded); no use of psychoactive medication or alcohol at bedtime.

Considering that we planned to perform separate, analogous analyses on the two groups (Recall and Recognition groups), we ran an a priori power analysis (through G*Power) with 1 group, 4 measurements, alpha = 0.05, rmANOVA, within factors, which indicated that a sample of 24 subjects (per group) was necessary to obtain an effect size of 0.04 with 80% power. During the time allotted for recruitment, 65 volunteers were screened in order to obtain a total sample of 48 participants. Twenty‐five potential participants had to be excluded because of the presence of sleep disorders or poor sleep quality (PSQI score > 5), the presence of anxiety and/or depressive symptoms (BAI score > 25, BDI‐II score > 29), and regular use of alcohol in the evening. Therefore, the final sample was made up of 40 individuals (20 M, 20 F; mean age = 22.5 ± 1.9) who were randomly assigned to two gender‐matched groups.

All participants provided informed consent and received no money or credit compensation. The study was submitted to the Ethical Committee of the University of Campania ‘L. Vanvitelli’ which approved the research (code 22/2020) and certified that the involvement of human participants was performed according to acceptable standards.

### 2.2 Procedure

All participants were administered a learning and a delayed test phase of the DRM paradigm under two within‐subjects conditions, ‘Sleep’ (retention period spent asleep) and ‘Wake’ (retention period spent awake), separated by a one‐week interval. The order of conditions was balanced between subjects.

The procedure for the two groups was identical except for the nature of the DRM test phases: free recall in the Recall Group and recognition in the Recognition Group (see below, ‘DRM task’ paragraph).

In Sleep, participants' nocturnal sleep was home‐monitored using the Dreem Headband 2 (DH; Dreem SAS, Paris), a portable sleep recording device. On the day before the sleep recording, participants were delivered the DH device and trained on how to wear it correctly. On the day scheduled for the recording, participants were administered the learning phase of the DRM task at 9 PM (± 1 h) and then wore the DH throughout the night. The test phase was performed 12 h after the learning phase, namely at 9 AM (± 1 h). Both the learning and the test phases were administered through a video call with the experimenter using the Google Form platform for the collection of responses.

On the day scheduled for Wake, participants performed the DRM learning phase at 9 AM (± 1 h) and the test phase at 9 PM (± 1 h), with the same procedure (video calls with the experimenter). During the retention interval, participants were allowed to engage in cognitively non‐demanding activities, which they later reported in a questionnaire, and were requested to avoid naps.

All learning and test sessions were performed at the subject's home. Subjects were asked to avoid the assumption of alcohol or other psychoactive substances and to maintain their regular sleep schedule on the days scheduled for experimental conditions as well as for the 2 days prior to each condition. To control for these factors, participants were also requested to fill in a sleep diary on the same days.

### 2.3 DRM Task

A slightly modified version of the DRM paradigm (Deese 1959; Roediger and McDermott 1995) was adopted to investigate false memory production in both groups. In the learning phase, participants learned 16 DRM word lists, 8 in Sleep and 8 in Wake. Each list consisted of 15 words (‘studied’ words) all semantically related to a critical ‘lure’ word that was not presented (e.g., ‘bed’, ‘rest’, ‘nap’ with ‘sleep’ as the lure). As in Roediger and McDermott (1995) and Iacullo and Marucci (2016), the words in each list were presented in order of associative strength with the unpresented lure (from strongest to weakest). Word lists were selected from the Italian standardised set by Iacullo and Marucci (2016); they were assigned to the Sleep and Wake conditions in balanced order between subjects.

As for task administration, the learning phase was identical between the two groups: the experimenter instructed participants to memorise the words as accurately as possible and then read the lists aloud. Immediate testing was then performed for both groups on 4 of the 8 presented lists (lists 2, 4, 6, 8). The selection of word lists for immediate testing was also balanced between participants. The two groups differ in the type of task adopted for testing (both immediate and delayed).

For the Recall Group, immediate testing consisted of a free recall task administered at the end of the presentation of each ‘Immediate Testing List’ (ITL list): as soon as the experimenter had read the list, participants were asked to type, on a blank page of the Google Form platform, all the words they remembered from that list (2 min allotted). The experimenter then proceeded to read the subsequent list and so on. As for delayed testing, performed 12 h later, participants were instructed to write, on a blank page of the Google Form platform, any words they remembered from all the presented lists (i.e., the 4 ITLs as well as the remaining 4 ‘No Immediate Testing Lists’, NITLs). They had 10 min to complete this task and were notified when they had 2 min remaining.

For the Recognition Group, testing consisted of a recognition test. Immediate testing was performed just after the presentation of each ITL. Specifically, the experimenter read aloud a list of 15 words, and participants were asked to verbally indicate whether each word was ‘old’ or ‘new’ (the experimenter proceeded to read the next word of the list as soon as the participant provided an answer to the previous). For each ITL, the 15 words of the immediate test corresponded to 8 studied words from that list (serial positions in the list: 1, 3, 5, 7, 9, 11, 13, 15), the lure word of that list, plus 6 unrelated words extracted from DRM lists not employed in the present study. The order of the 15 words was counterbalanced between subjects. As for the delayed test, participants were presented with a list of 120 words (in randomised order), made up of the 8 lure words, 30 studied words from the ITLs (serial positions 2, 4, 6, 8, 10, 12, 14), 30 studied words from the NITLs (serial positions 1, 3, 5, 7, 9, 11, 13, 15), and 52 unrelated distractors. Again, the experimenter read each of the 120 words aloud, and participants were asked to judge each word as ‘old’ or ‘new’.

### 2.4 Sleep Recordings

The DH is a wireless device that records, stores, and automatically analyses physiological data in real time without the need for connections (e.g., Bluetooth or Wi‐Fi). Physiological signals are recorded via three types of sensors: (1) brain cortical activity through 5 EEG electrodes yielding seven derivations (FpZ‐O1, FpZ‐O2, FpZ‐F7, F8‐F7, F7‐O1, F8‐O2, FpZ‐F8; 250 Hz with a 0.4–35 Hz bandpass filter); (2) movements, position, and breathing frequency through a 3D accelerometer located over the head; (5) heart rate via a red‐infrared pulse oximeter located in the frontal band. It provides an accurate automatic sleep staging classification (Arnal et al. 2020) that follows the American Academy of Sleep Medicine guidelines (AASM; Iber et al. 2007).

From the DH recording, we extracted:

– Time in bed (TIB, i.e., total amount of time, in minutes, from lights off to final awakening);
– Total sleep time (TST, i.e., total amount of time, in minutes, from the first appearance of N1 to final awakening);
– Sleep‐onset latency in minutes (SOL);
– Wake after sleep onset in minutes (WASO);
– Sleep efficiency (SE%, i.e., percentage of TST over TIB);
– Sleep stage proportions over TST (N1%, N2%, N3%, REM%).

### 2.5 Performance Measures

For the Recall Group, outcome measures of immediate and delayed performance at the DRM task were:

– Number of False Recalls (i.e., total number of falsely recalled lure words);
– Number of Veridical Recalls (i.e., total number of studied words correctly recalled);
– Number of Related intrusions (i.e., total number of falsely recalled words not corresponding to the lure words but semantically related to them);
– Number of Unrelated intrusions (i.e., total number of falsely recalled words not corresponding to the lure words and not semantically related to them);
– Proportion of False Recalls, i.e., number of False Recalls in proportion to the total number of lures;
– Proportion of Veridical Recalls, i.e., number of Veridical Recalls in proportion to the total number of studied words.

As for the Recognition Group, we measured:

– False Recognition Rate (FRR), i.e., the proportion of ‘old’ responses given to lures over the total number of lures;
– True Recognition Rate (TRR), i.e., the proportion of ‘old’ responses given to studied words over the total number of studied words (i.e., ‘hits’);
– False Alarm Rate (FAR), i.e., the proportion of ‘old’ responses given to distractors over the total number of distractors (i.e., ‘false alarms’).

For both groups, performance measures at the delayed test were calculated separately for ITLs and NITLs, except for intrusions and FAR, since intrusions and false alarms cannot be attributed to any specific list (either subjected to immediate testing or not).

### 2.6 Data Analysis

To control that baseline measures did not differ between conditions, we compared immediate testing performance of Sleep and Wake through the Wilcoxon signed‐rank test for the Recall Group (due to non‐normal distributions of the data) and paired‐samples t‐tests for the Recognition Group.

For both groups, delayed performance was assessed through linear mixed models (LMM), using DRM outcome measures (except intrusions and FAR) as dependent variables, ‘Condition’ (Sleep and Wake) and ‘List‐type’ (ITL and NITL) as fixed factors, participants’ ID as a random effect, and the factor ‘Immediate Testing’ as a covariate. We also included in the model the interactions between Immediate Testing and the other variables. As for intrusions and FAR, the same analysis was carried out with the same factors excluding List‐type, since intrusions and false alarms are not univocally attributable to any specific list (either subjected to immediate testing or not). Furthermore, for intrusions, ‘Intrusion‐type’ (Related and Unrelated) was included as a fixed factor.

Holm correction was adopted for post hoc tests, and the corrected p‐value is reported. Moreover, Pearson's correlation analysis was performed to assess the relationship between delayed performance measures of Sleep and the sleep measures collected in the same condition.

Finally, we compared memory performance between groups by means of a LMM analysis, with false memories (proportion of False Recalls and FRR) and veridical memories (proportion of Verifical Recalls and TRR) as dependent variables, ‘Condition’ (Sleep and Wake), ‘List‐Type’ (ITL and NITL) and ‘Test‐type’ (Recall and Recognition) as fixed factors, participants’ ID as a random effect, and the factor Immediate Testing as acovariate.

Note that including the order of conditions (Sleep and Wake) in the LMMs did not result in any significant effects or changes in the main results. Therefore, for clarity purposes, here we report results of a simplified LMM which does not take into account the order of conditions.

Analyses were performed using JAMOVI (2.5.6.0) and the significance level was set at *p* < 0.05.

## 3 Results

Two subjects had to be excluded from the analyses of the Recall Group: one participant slept < 4 h and another did not wear the DH during the sleep recording night. Thus, the final results for the Recall Group are derived from data collected on 18 subjects (10 F; mean age = 22.51 ± 1.88 years), whereas those for the Recognition Group are drawn from 20 subjects (10 F; mean age = 22.45 ± 2.43 years). Table 1 displays the characteristics of these two groups in terms of circadian preference, anxiety and depression symptoms, habitual subjective sleep quality, and insomnia symptoms. No significant differences were observed between groups.

**TABLE 1:** Questionnaire scores in the two groups.

	Recall group	Recognition group	*t*	*p*	Cohen's d
MEQr	13.56 ± 4.11	13.20 ± 4.18	0.264	0.793	0.086
BAI	10.50 ± 8.53	9.90 ± 7.89	0.225	0.823	0.073
BDI‐II	10.06 ± 8.59	8.25 ± 7.34	0.699	0.489	0.227
PSQI	4.78 ± 1.96	5.10 ± 2.43	−0.780	0.441	−0.253
ISI	4.94 ± 3.84	4.95 ± 3.82	−0.004	0.996	−0.001

*Note*: Data are reported as means ± standard deviations.

Abbreviations: BAI = Beck Anxiety Inventory; BDI‐II = Beck Depression Inventory‐II; ISI = Insomnia Severity Index; MEQr = Morningness‐Eveningness Questionnaire reduced version; PSQI = Pittsburgh Sleep Quality Index.

### 3.1 Sleep Measures

Table 2 displays sleep measures collected through the DH device in the Sleep condition in the two groups. The only significant difference was observed for SOL, with longer SOL in the Recall Group. No significant between‐groups difference emerged for the other sleep parameters.

**TABLE 2:** Sleep measures (Sleep condition) in the two groups.

Sleep measures	Recall group	Recognition group	*t*	*p*	*Cohen's d*
Bedtime (hh:mm)	00:12 ± 01:15	00:38 ± 00:56	−1.19	0.241	−0.39
TIB (min)	448.63 ± 68.71	453.76 ± 46.56	−0.272	0.787	−0.88
TST (min)	410.58 ± 64.48	422.57 ± 55.65	−0.615	0.542	−0.200
SOL (min)	22.09 ± 14.48	13.42 ± 11.07	2.090	**0.044**	0.679
WASO (min)	15.96 ± 17.94	17.78 ± 297.82	−0.225	0.824	−0.073
SE%	93.33 ± 3.81	94.35 ± 7.61	−0.516	0.609	−0.168
N1%	0.46 ± 0.57	0.40 ± 0.57	0.357	0.723	0.116
N2%	47.80 ± 9.19	45.32 ± 9.61	0.811	0.423	0.263
N3%	28.78 ± 7.95	27.82 ± 7.33	0.390	0.699	0.127
REM%	24.86 ± 5.13	27.46 ± 6.40	−1.371	0.179	−0.446

*Note*: Significant *p* values are in bold.

Abbreviations: SE = Sleep Efficiency; SOL = Sleep‐Onset Latency; TIB = Time in Bed; TST = Total Sleep Time; WASO = Wake After Sleep Onset.

### 3.2 Immediate Recall and Recognition Performance

As displayed in Table 3, no significant differences between conditions emerged for any of the baseline performance measures in either the Recall or Recognition groups.

**TABLE 3:** Comparison of baseline measures of DRM performance (Immediate Testing) between Sleep and Wake in the two groups.

	Sleep	Wake	Z	*p*	ES
Recall Group					
False recalls	1.28 ± 1.18	1.28 ± 1.18	52.00	0.999	−0.010
Veridical recalls	29.94 ± 28.50	30.38 ± 30.00	64.00	0.358	−0.251
Related intrusions	1.23 ± 1.93	0.78 ± 1.06	7.50	0.461	0.500
Unrelated intrusions	0.33 ± 0.84	0.11 ± 0.47	55.00	0.524	0.21

	Sleep	Wake	*t*	*p*	Cohen's d
Recognition group					
False recognition rate	0.74 ± 0.30	0.65 ± 0.36	1.29	0.273	0.252
True recognition rate	0.88 ± 0.07	0.84 ± 0.07	1.38	0.183	0.309
False alarm rate	0.06 ± 0.05	0.07 ± 0.08	−0.11	0.910	−1.521

*Note*: Z = Wilcoxon's Z; t = Student's t. Means ± standard deviations are reported.

### 3.3 Delayed Recall Performance (Recall Group)

**FIGURE 1:**
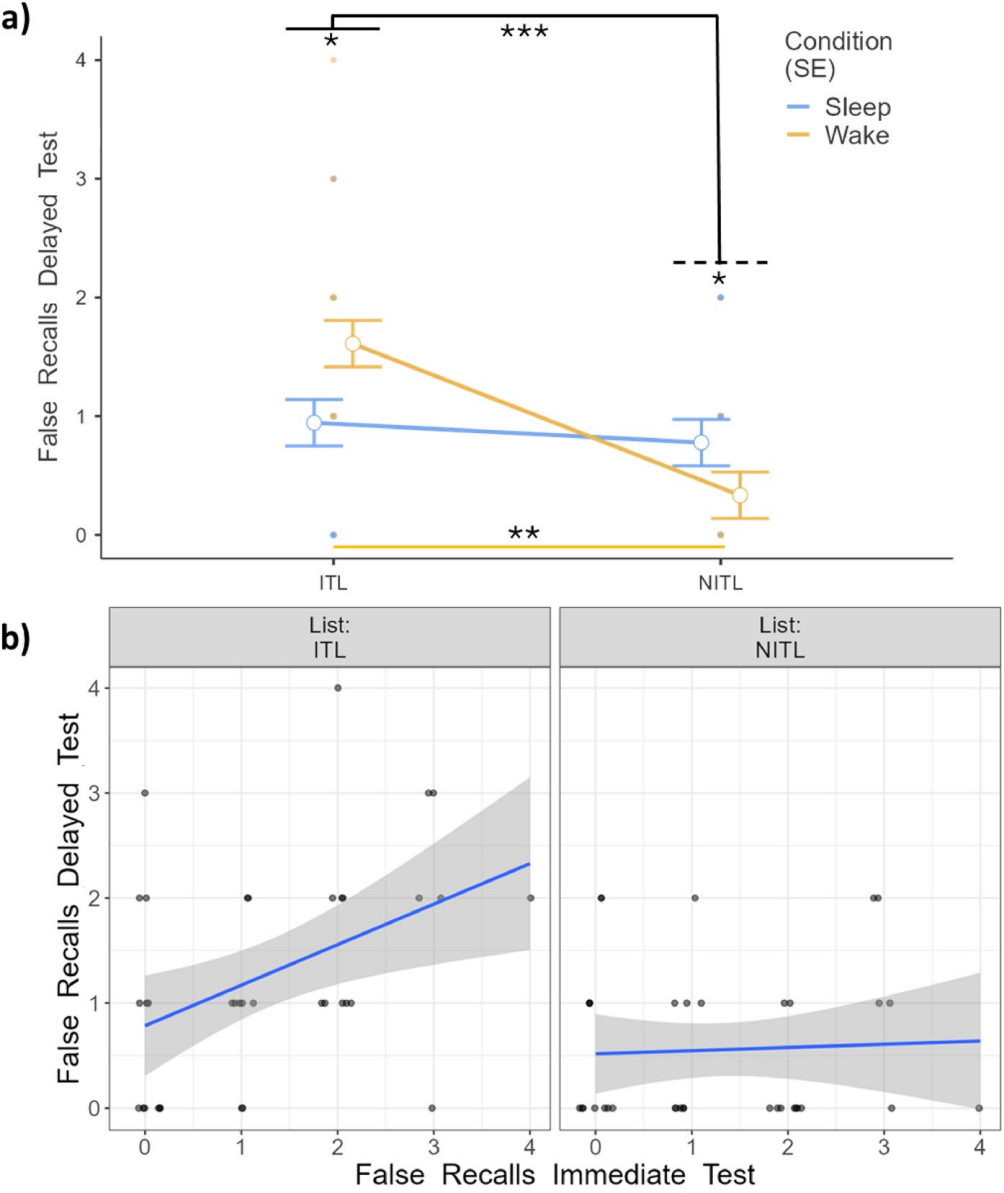
(a) False Recalls at the delayed test as a function of condition (Sleep vs. Wake) and type of list (ITLs vs. NITLs). (b) False Recalls as a function of performance at the immediate test and of the type of list. Each dot represents a participant. ITL: Immediate Testing List. NITL: No Immediate Testing List. SE: Standard Error of the mean. *p < 0.05; **p < 0.01; ***p < 0.001.

Mixed models on False Recalls revealed a significant effect of List‐type (*F*_1,47.6_ = 16.54, *p* < 0.001) and Immediate Testing (*F*_1,59.9_ = 4.83, *p* = 0.032), indicating that ITLs more frequently induced subjects to falsely recall lure words than NITLs and that the more False Recalls were produced at the immediate test, the more were recalled at the delayed test. The interaction Condition × List‐type (*F*_1,47.6_ = 9.79, *p* = 0.003; Figure 1a) was significant. Specifically, participants produced more False Recalls at ITLs in Wake than in Sleep (*t* (47.3) = − 2.67, *p* = 0.043), whereas False Recalls at NITLs were more numerous in Sleep than in Wake (*t* (47.3) = 1.77, *p* = 0.016). Moreover, in Wake, the number of False Recalls was higher for ITLs than for NITLs (*t* (47.3) = 3.32, *p* = 0.009). The List‐type × Immediate Testing interaction (*F*_1,47.6_ = 5.25, *p* = 0.026) was also significant, indicating that the more False Recalls were produced at the immediate test (ITLs), the more the same were retrieved at the delayed test (*t* (47.6) = − 2.29, *p* = 0.026, Figure 1b). The effect of Condition (*F*_1,47.6_ = 0.39, *p* = 0.535), as well as the interaction Condition × List‐type × Immediate Testing (*F*_1,47.6_ = 0.368, *p* = 0.547) and the interaction Condition × Immediate Testing (*F*_1,55.5_ = 2.52, *p* = 0.118) were not significant.

**FIGURE 2:**
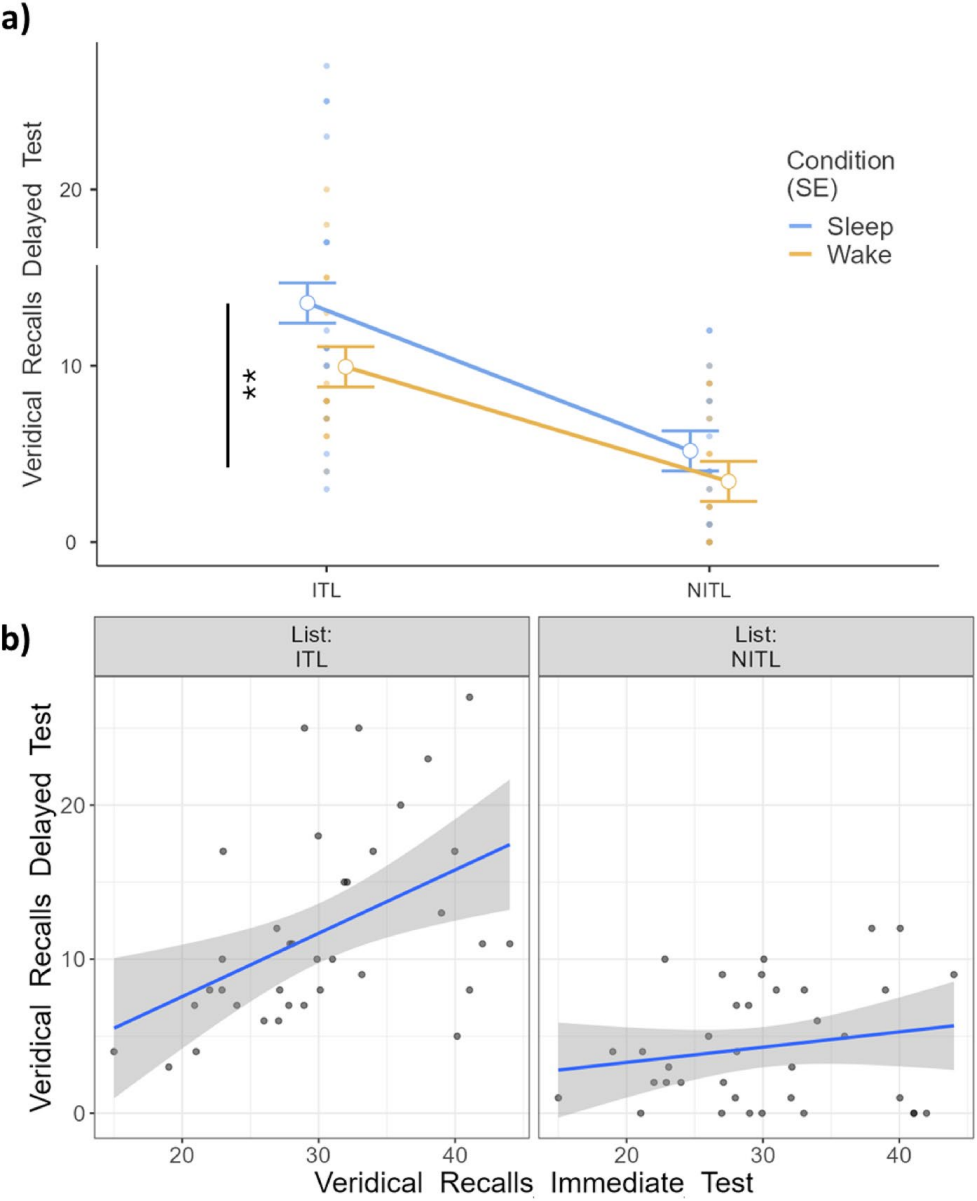
(a) Veridical Recalls at the delayed test as a function of condition (Sleep vs. Wake) and type of list (ITLs vs. NITLs). (b) Veridical Recalls as a function of performance at the immediate test and of the type of list. Each dot represents a participant. ITL: Immediate Testing List. NITL: No Immediate Testing List. SE and shaded areas: Standard Error of the mean. **p < 0.01.

As for Veridical Recalls, we observed a significant effect of Condition (*F*_1,48.1_ = 7.57, *p* = 0.008), with participants correctly recalling more studied words in Sleep compared to Wake (Figure 2a). Moreover, a significant effect of List‐type emerged (*F*_1,48_ = 59.21, *p* < 0.001), with ITLs more frequently inducing Veridical Recalls compared to NITLs. The effect of Immediate Testing was also significant (*F*_1,42.3_ = 8.38, *p* = 0.006), indicating that the more studied words were recalled at the immediate test, the more were correctly recalled at the delayed test. Finally, we observed a significant List‐type × Immediate Testing interaction (*F*_1,48_ = 4.12, *p* = 0.048), suggesting that the more veridical recalls were produced at the immediate test, the more the same were retrieved at the delayed test. The interactions Condition × List‐type (*F*_1,48_ = 1.07, *p* = 0.305) and Condition × List‐type × Immediate Testing (*F*_1,48_ = 1.06, *p* = 0.309) were not significant.

Finally, analyses on intrusions showed a significant main effect of Condition (*F*_1,52.8_ = 7.07, *p* < 0.010) and an interaction Condition × Intrusion‐Type (*F*_1,55.6_ = 14.09, *p* < 0.001), with participants reporting more Unrelated intrusions in Wake compared to Sleep (*t* [51.1] = 6.22, *p* < 0.001). Also, in Sleep there was no significant difference between Unrelated and Related Intrusions (*t* [51.1] = −1.73, *p* = 0.159), whereas in Wake participants reported more Unrelated than Related Intrusions (*t* [56.6] = 3.44, *p* = 0.003). Immediate testing was a significant covariate for all of the factors (all F's > 12.79, all *p*'s < 0.001), indicating that participants reporting more intrusions at the immediate test also reported more intrusions at the delayed test, and this was especially true in Wake and for Unrelated Intrusions (see Figure S1).

### 3.4 Delayed Recognition Performance (Recognition Group)

**FIGURE 3:**
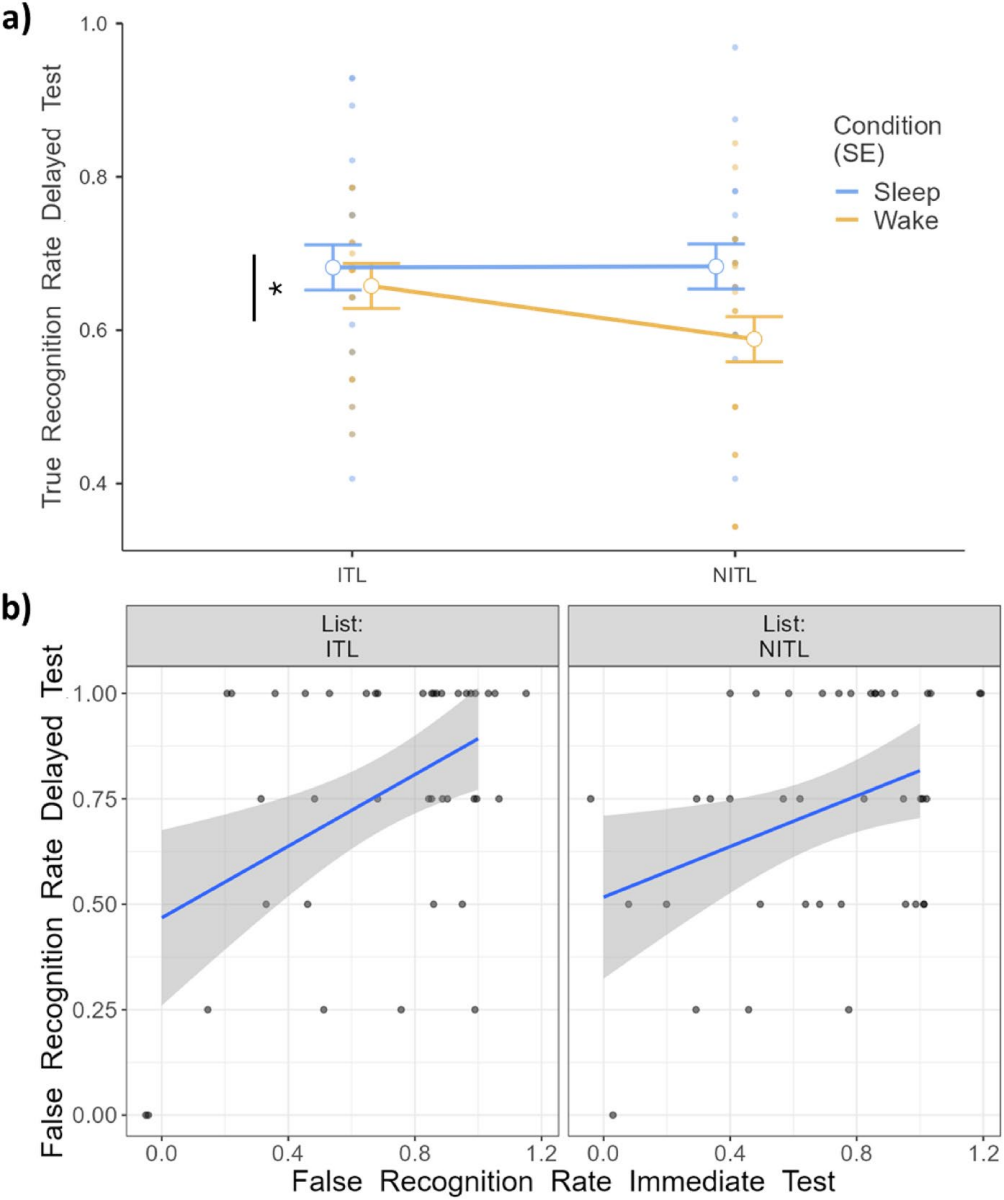
(a) True Recognition Rate at the delayed test as a function of condition (Sleep vs. Wake) and type of list (ITLs vs. NITLs). (b) False Recognition Rate as a function of performance at the immediate test and of the type of list. Each dot represents a participant. ITL: Immediate Testing Lists. NITL: No Immediate Testing Lists. SE and shaded areas: Standard Error of the mean. *p < 0.05.

Analyses on FRR revealed a significant effect of Immediate Testing (*F*_1,51_ = 5.02, *p* = 0.029), indicating that subjects showing high FRR at the immediate test more frequently recognised the lure words as old at the delayed test, independent of study condition (Figure 3b). No effect of Condition (*F*_1,52.6_ = 0.01, *p* = 0.940) or List‐type (*F*_1,51_ = 0.27, *p* = 0.599) emerged. The interactions Condition × List‐type (*F*_1,50.6_ = 2.67, *p* = 0.108), Condition × Immediate Testing (*F*_1,65.7_ = 1.04, *p* = 0.311), List‐type × Immediate Testing (*F*_1,50.6_ = 1.57, *p* = 0.215) and Condition × List‐type × Immediate Testing (*F*_1,50.6_ = 2.18, *p* = 0.143) were also non‐significant.

As for TRR, we observed a significant effect of Condition (*F*_1,54.4_ = 6.38, *p* = 0.014), with participants correctly recognising more studied words in Sleep compared to Wake (Figure 3a). No effect of List‐type (*F*_1,52.7_ = 1.18, *p* = 0.281) or Immediate Testing (*F*_1,71_ = 0.72, *p* = 0.399) emerged. The interactions Condition × Immediate Testing (*F*_1,71.9_ = 0.31, *p* = 0.579), List‐type × Immediate Testing (*F*_1,52.7_ = 1.46, *p* = 0.232), Condition × List‐type (*F*_1,52.7_ = 3.19, *p* = 0.079) and Condition × List‐type × Immediate Testing (*F*_1,52.7_ = 2.74, *p* = 0.104) were also not significant.

Finally, as for FAR, we observed a significant effect of Immediate Testing (*F*_1,35_._5_ = 9.06, *p* = 0.005), indicating that the higher the FAR at the immediate test, the higher the FAR at the delayed test. No effect of Condition (*F*_1,16.2_ = 0.01, *p* = 0.543) and no Condition × Immediate Testing interaction (*F*_1,20.8_ = 0.16, *p* = 0.692) emerged.

### 3.5 Comparison Between Recall and Recognition Performance

**FIGURE 4:**
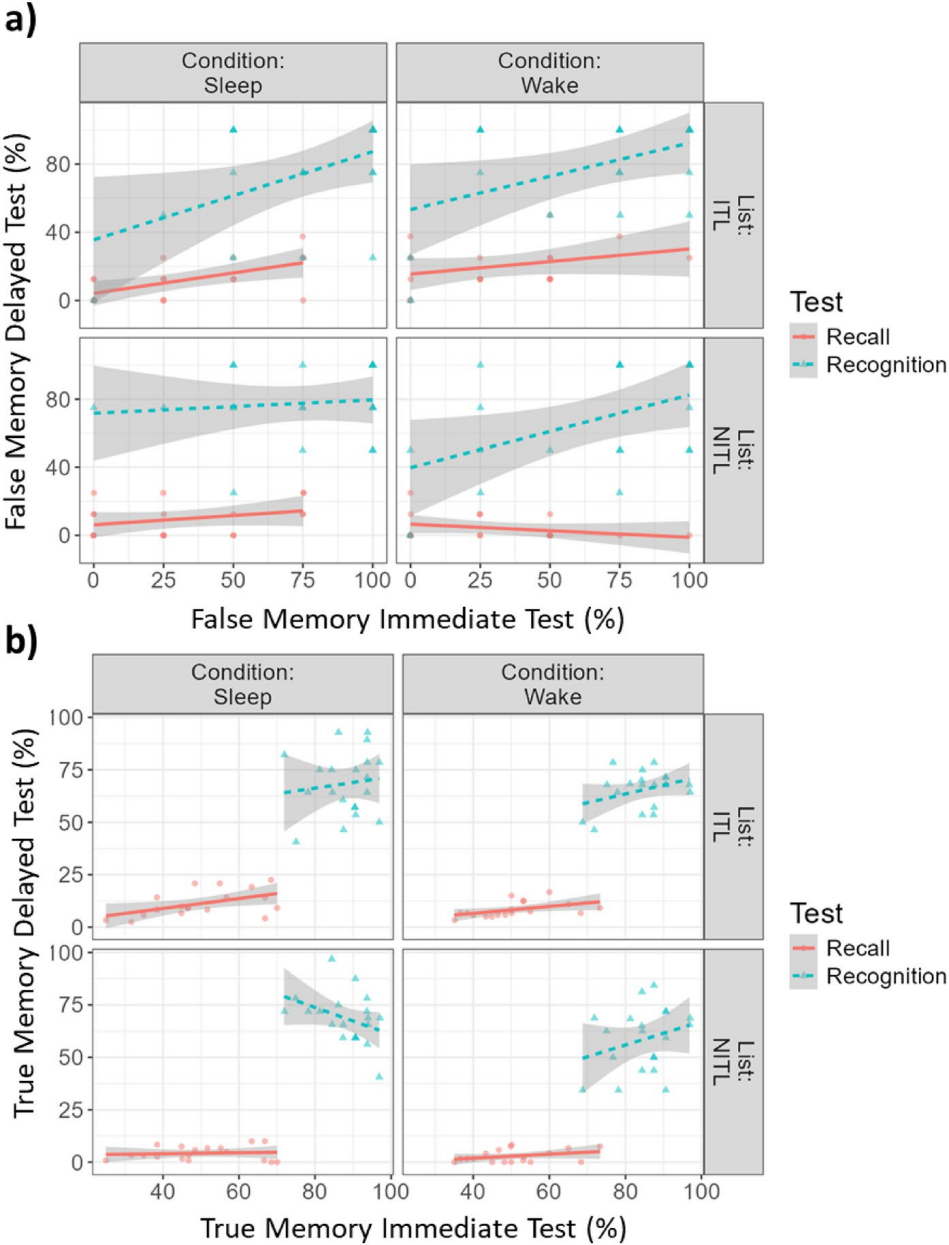
Comparison between recall and recognition performance. Percentage of (a) False Memory and (b) True Memory as a function of the Condition (Sleep vs. Wake), of the List (ITL vs. NITL), the Task (Recall vs. Recognition), and the Immediate Test performance. ITL: Immediate Testing List. NITL: No Immediate Testing List. Shaded areas: Standard error of the mean.

The LMM on false memory variables (Figure 4a) showed a significant main effect of Test type (*F*_1,41.10_ = 121.45, *p* < 0.001), showing a higher proportion of false memories at the Recognition compared to the Recall task, a significant effect of List type (*F*_1,103.4_ = 4.80, *p* = 0.031), with more false memories produced from ITLs than NITLs, as well as a significant main effect of Immediate Testing (*F*_1,126.43_ = 8.80, *p* = 0.004). The LMM also showed a significant interaction Condition × List type (*F*_1,103.41_ = 6.13, *p* = 0.015): specifically, Holm test revealed a significant difference between List types in Wake, with higher probability of a false memory for items of ITLs than NITLs. All other effects were not significant (all *F*'s < 0.82, all *p's* > 0.368).

The LMM on true memory variables (Figure 4b) showed a main effect of Test type (*F*_1,96.5_ = 168.10, *p* < 0.001), with a higher probability of correctly remembering items in the Recognition Task, a significant effect of List type (*F*_1,104.9_ = 15.50, *p* < 0.001), with more items correctly remembered from ITLs, and a significant main effect of Condition (*F*_1,106.79_ = 9.64, *p* < 0.001), with a higher percentage of correctly remembered items in Sleep. All other effects were not significant (all F's < 3.82, all *p's* > 0.053).

### 3.6 Correlations Between DRM Delayed Performance and Sleep Measures

As for the Recall Group, no significant correlation emerged between DRM delayed performance and sleep measures.

The same was true for the Recognition Group, except for a weak and uncorrected positive correlation observed between FAR and N3% (*r* = 0.44, *p* = 0.050).

## 4 Discussion

In this study, we addressed two methodological issues concerning the literature on sleep‐related effects on false memory formation, i.e., (a) the inconsistent evidence on whether sleep promotes false memories only when tested through free recall rather than recognition tasks, and (b) the absence in available studies of immediate testing procedures allowing us to ascertain whether false memory production after sleep compared to wake depends on memory processes unfolding during the retention interval rather than on between‐conditions differences at encoding. Indeed, as far as we are aware, this is the first study to investigate sleep‐related false memory performance through a free recall and a recognition task in two homogeneous, age‐matched samples and to compare delayed false memory performance preceded or not by immediate testing in a within‐subjects design.

In both groups we observed no differences in baseline DRM performance (including both false and veridical memories) between the Sleep and Wake conditions, notably allowing us to support the hypothesis that delayed performance findings depend on offline memory reprocessing during the retention interval.

Moving on to delayed performance, a first important remark regards the different pattern of findings observed in the Recall vs. the Recognition Group, which confirms previous literature on sleep‐related false memory formation. In fact, whereas effects of condition emerged for False Recalls (Recall Group), no between‐conditions differences were found for FRR (Recognition Group). Specifically, if we compare our results on NITLs (word lists not subjected to immediate testing) of both groups to those of previous studies conducted without immediate testing, our data overlap with extant findings and confirm that sleep relative to wake promotes false memory formation when tested through a free recall procedure but not through a recognition task. As previously proposed (e.g., Pardilla‐Delgado and Payne 2017; Newbury and Monaghan 2019; Malloggi et al. 2022, 2023), this finding can be explained by the different cognitive demands entailed by the different nature of the tasks: whereas in the recognition task subjects are required to discriminate between ‘old’ and ‘new’ words, the free recall task does not provide any external memory cues for retrieval (subjects are required to generate their own cues to reinstate the original memory traces). This process of ‘self‐cueing’ is more cognitively demanding and entails a higher risk of memory errors compared to the recognition procedure, which is instead more likely to promote source monitoring (Pardilla‐Delgado and Payne 2017). Given that sleep is known to improve source monitoring abilities in the subsequent wake period (Johnson et al. 1993), the sleep‐related consolidation of false memories observed in free recall paradigms would be compensated by the enhanced source monitoring ability in recognition paradigms, explaining the lack of differences between sleep and wake conditions in this latter type of paradigm. Incidentally, this hypothesis is also compatible with the other literature results showing reduced false recognition after sleep relative to wake intervals (Fenn et al. 2009; Lo et al. 2014).

Another relevant finding is that delayed free recall performance is critically influenced by the presence of an immediate test, as shown by the significant effects of the factor List‐type found both for false and veridical memories. Indeed, the presence of immediate testing increased both false and veridical free recall after the retention interval compared to memories for lists that had not been previously tested. This is not particularly surprising: it is plausible, in fact, that the repetition of the lists subjected to immediate testing during the learning phase has increased trace strength, both for falsely recalled lures and for correctly remembered words. The fact that this effect was not observed for the production of false alarms at the recognition task is probably explainable through a similar reasoning as the one exposed above: the less demanding nature of the recognition task would promote more accurate source monitoring processes at retrieval independent of original trace strength. Similarly, a ceiling effect may have masked the effect of immediate testing on the recognition of studied words. Still, significant main effects of the covariate Immediate Testing (indicating that the immediate test enhanced the probability of recalling or recognising the same kind of words at the delayed test) emerged for most performance variables, both in the recall and in the recognition group, suggesting that effects of trace strength influence also recognition processes.

Particularly interesting is the pattern of data on false memories produced at free recall. Indeed, the presence of an immediate test appears to have different effects on delayed false recall performance based on the behavioural state in which the retention interval took place, as indexed by the significant interaction observed between Condition and List‐type. Specifically, after sleep, the number of false recalls produced was slightly lower than that reported at immediate testing, and it was similar for both types of lists (those subjected and those not subjected to immediate testing). Instead, in Wake, the number of false recalls from previously tested lists was significantly higher than that from lists not previously tested. As for between‐conditions comparisons, participants produced more false recalls at tested lists in Wake than in Sleep, whereas false recalls at the NITLs were more numerous in Sleep than in Wake. Furthermore, it is noteworthy that the false memories produced at the delayed recall of ITLs mostly corresponded to those produced at immediate recall in both Sleep and Wake. Taken together, these results induce us to reconsider the interpretation given to our previous observations (Malloggi et al. 2023), as well as to similar literature findings (e.g., Payne et al. 2009; Diekelmann et al. 2010) showing greater false memory production after sleep relative to wake at free recall tasks not preceded by immediate testing. In fact, this pattern of data has been repeatedly attributed to sleep's role in promoting processes of gist abstraction. This hypothesis would lead to the prediction that new false memories would emerge after the sleep period, a prediction that is not supported by our findings. Instead, our data suggest that, as occurs for veridical memories, sleep protects false memories produced at encoding from decay, irrespective of the presence of immediate testing, whereas, when the retention interval is spent awake, the presence or absence of an immediate test yields differential outcomes on delayed false memory production. In fact, it appears that, in the absence of immediate testing, false memories formed at encoding undergo greater decay during wake relative to sleep (as in the case of veridical memories), while their recall is increased in the Wake compared to the Sleep condition when they have undergone immediate reproduction. A possible interpretation of this latter observation refers to the concept of ‘reconsolidation’ (Nader 2015), whereby the reactivation of a memory trace makes it transiently unstable, i.e., more susceptible to interference and disruption. This process could have plausibly occurred in Wake (thus explaining why false recalls from ITLs were higher than those from NITLs in this condition), whereas it would have been counteracted by the enhancement of source monitoring ability (Johnson et al. 1993) in Sleep. Our findings on intrusions produced at free recall are also coherent with these hypotheses. Indeed, intrusions were more numerous in Wake than in Sleep (in line with Mak et al. 2023). Specifically, participants produced significantly more unrelated intrusions (i.e., intrusions which were semantically unrelated to any of the list themes) in Wake than in Sleep, suggesting, again, lower source monitoring ability after the waking retention interval. Moreover, participants reporting more intrusions at the immediate test also reported more intrusions at the delayed test, and this was especially true in Wake and for unrelated intrusions.

Of note, the results on false memory proportions (i.e., from our analyses including both groups) are consistent with the findings and hypotheses discussed above. In fact, although some effects of Condition failed to reach significance, the analysis highlighted an interaction between Condition and List‐Type. Specifically, it showed that, irrespective of the type of task (free recall or recognition), false memories from tested lists were more numerous than those from unpracticed lists only in Wake.

As for veridical memories, our results highlight that sleep, relative to wake, protects the studied words from decay both in the free recall and the recognition task, regardless of the presence of immediate testing. This finding is in line with previous studies adopting the DRM paradigm (Payne et al. 2009; McKeon et al. 2012; Pardilla‐Delgado and Payne 2017; Malloggi et al. 2023), as well as with the more general, robust literature on sleep and declarative memory (for a review, see Conte and Ficca 2013). These results are further confirmed by our between‐groups comparisons, which showed that veridical memories were more numerous in the recognition task compared to the free recall task, for ITLS rather than NITLs (independently of condition and type of test) and in Sleep compared to Wake (independently of type of test and presence of the immediate test).

An important additional observation regards the possible interpretation of our results in light of existing theoretical accounts on retrieval‐induced forgetting (RIF), i.e., the phenomenon of retrieval practice causing non‐practiced items to become less recallable (see Murayama et al. 2014 for a review). In a typical RIF research paradigm, participants study items from different semantic categories and then repeatedly retrieve half of the items from half of the categories; when memory for all items is tested, recall of unpracticed items from practiced categories is usually poorer than recall of items from unpracticed lists (Murayama et al. 2014). When considering our findings in light of this literature, we cannot exclude that the retrieval of some items at the immediate test has interfered with delayed retrieval of items of the same lists that had not been previously retrieved. However, our finding that, in the Recall group, non‐tested lists were recalled more poorly than tested lists in both conditions does not support this hypothesis. Similarly, our results on false recalls and FRR are in contrast with those of Starns and Hicks (2004), who observed that false recall and false recognition of critical lures associated with practiced lists were lower than false recall and false recognition of lures associated with unpracticed lists. Still, it must be underlined that comparisons of our data to those from studies on the RIF are to be taken cautiously because of the significant methodological differences, regarding especially the nature of the memory task. In fact, in order to have greater control on which specific items undergo retrieval practice, studies on the RIF generally employ word stem completion tasks in the practice phase and cued recall as the final performance test, rather than free recall procedures.

Based on our pattern of results, some conclusions and suggestions can be drawn on the use of IT in sleep‐memory studies. As highlighted by studies from psychologists of memory, of course, the introduction of an immediate test will in any case affect the consolidation process, e.g., by inducing RIF or reconsolidation mechanisms. However, at least as far as veridical memories are concerned, these effects seem to equally involve both the sleep and wake conditions. In line with this, we showed that veridical memories are enhanced by the immediate test independently of condition. These observations support the introduction of immediate testing as a standard procedure in classical sleep‐memory designs (comparing the effect of sleep and wake intervals on veridical recall), in light of its multiple advantages (as already argued in Conte and Ficca 2013; Németh et al. 2024): (a) provided that immediate performance does not differ between conditions, it allows ruling out that what is observed at delayed testing is a consequence of differential encoding effectiveness rather than consolidation processes; (b) it provides a measure of baseline performance, allowing determining the presence and extent of decay or enhancement of memories across the retention interval. Conversely, our results on false recalls, with the immediate test affecting performance across wake but not sleep intervals, suggest that its use should undergo a much more careful choice in studies on sleep and false memory.

A few limitations of the study should be acknowledged. The generalizability of our findings is limited by our sample size, which is lower than what is recommended by power analysis. Still, a a posteriori power analysis (with 1 group, 4 measurements, alpha = 0.05, rmANOVA, within factors) indicated that a sample of 20 subjects per group is enough to obtain an effect size of 0.04 with 70% power. Moreover, a technical limitation should be taken into account when considering the objective sleep parameters we collected in Sleep. In fact, while automatic sleep scoring performed with the Dreem Headband (compared to traditional polysomnographic devices) has proven sufficiently accurate (Arnal et al. 2020), its data have proven unreliable when addressing EEG spectral components (Mallender and Depner 2023; Ravindran et al. 2024). Therefore, the classification of sleep states (especially NREM substates) carried out with the Dreem Headband should be considered cautiously. For the same reasons, we chose not to analyse some relevant sleep architecture parameters, including, e.g., spectral power or spindle activity and slow oscillations, which could have provided greater insights into underlying mechanisms. Nevertheless, the Dreem Headband's greater practicality and comfort for the user allow for much higher feasibility of this type of research in shorter time periods.

In conclusion, our findings confirm, in two homogeneous, age‐matched samples, that sleep relative to wake enhances false recalls but not false recognitions when memory is tested directly after the retention interval. Moreover, we investigated the effect of immediate testing on delayed false memory performance. By comparing within‐groups delayed performance at previously tested and non‐tested word lists, we show that delayed performance can be assumed to be dependent on sleep (vs. wake) ‐related consolidation processes rather than on between‐conditions differences at encoding. Furthermore, we highlight that immediate testing enhances delayed veridical recall performance across both sleep and wake intervals, while its effect on delayed false recall depends on the behavioural state in which retention occurs.

These data bear important theoretical and applicative implications. On one hand, they question the common interpretation that sleep over wake enhances false memory formation through overnight facilitation of gist abstraction processes, suggesting, instead, that a period filled with sleep relative to a period of exclusive wakefulness protects the accuracy of memories produced at encoding (whether false or veridical). From an applicative standpoint, our findings bring attention to the choice of employing or avoiding immediate testing procedures in research designs comparing the effect of sleep and wake on false memory production. This choice should be accurately evaluated in relation to study objectives and data interpretation, considering the relevant effects that inclusion/exclusion of such a procedure bears on delayed performance results. Furthermore, our study prompts further research, conducted in more ecological contexts, on the formation and consolidation of false memories across sleep and wake periods. Indeed, in applicative fields of research such as that on eyewitness testimony, it may be important to bear in mind that sleeping or waking after the reinstatement of a critical memory can significantly influence the future outcome of that memory, with sleep facilitating its accuracy and wake rendering it more vulnerable to misinformation effects (Loftus 2005).

## Author Contributions

**Francesca Conte:** conceptualization, methodology, writing – original draft, writing – review and editing. **Serena Malloggi:** conceptualization, methodology, formal analysis, investigation, writing – original draft. **Oreste de Rosa:** formal analysis, investigation. **Gianluca Ficca:** conceptualization, writing – review and editing, supervision, project administration. **Fiorenza Giganti:** conceptualization, methodology, writing – review and editing, supervision, project administration. **Nicola Cellini:** conceptualization, methodology, formal analysis, writing – original draft, writing – review and editing, supervision, project administration.

## Conflicts of Interest

The authors declare no conflicts of interest.

## Supporting information


**Figure S1.** Intrusions at the Delayed Test as a function of performance at the Immediate Test, Intrusion‐Type (Unrelated vs. Related), and Condition (Sleep vs. Wake). Each dot represents a participant. Shaded areas: standard error of the mean.

## Data Availability

The data that support the findings of this study are available from the corresponding author upon reasonable request.
