# Supplementary material for: Sleep and False Memory Production: The Modulating Role of Immediate Testing and Type of Retrieval
Source: J Sleep Res. 2025 Mar 21;35(1):e70051. doi: 10.1111/jsr.70051 (PMC12856130; doi:10.1111/jsr.70051)
Supplement: Supplementary file 1 — Figure S1. Intrusions at the Delayed Test as a function of performance at the Immediate Test, Intrusion‐Type (Unrelated vs. Related), and Condition (Sleep vs. Wake). Each dot represents a participant. Shaded areas: standard error of the mean. [file JSR-35-e70051-s001.docx]

**Supplementary Figure**


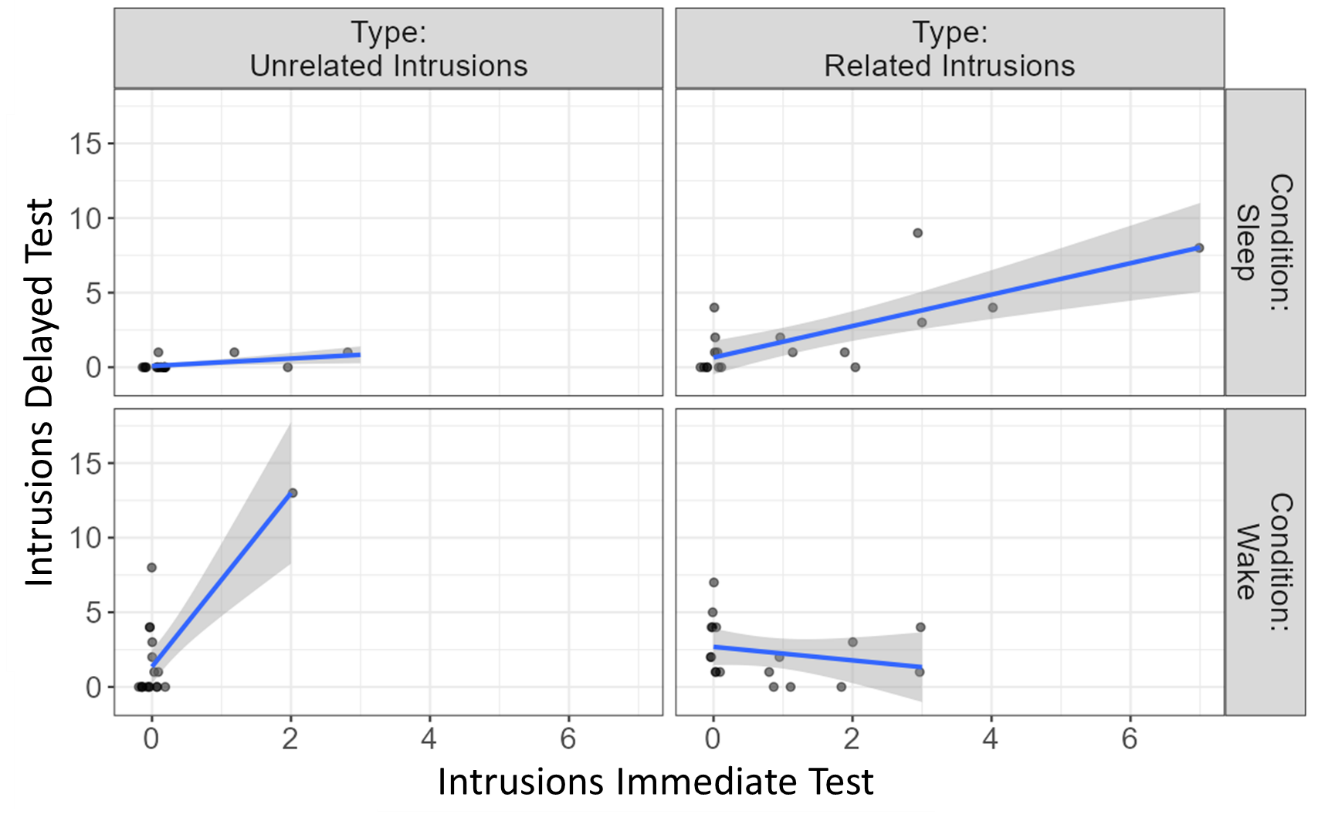


**Figure S1.** Intrusions at the Delayed Test as a function of performance at the Immediate Test, Intrusion-Type (Unrelated vs Related), and Condition (Sleep vs Wake). Each dot represents a participant. Shaded areas: standard error of the mean.
